# Elevated galanin receptor type 2 primarily contributes to mechanical hypersensitivity after median nerve injury

**DOI:** 10.1371/journal.pone.0199512

**Published:** 2018-06-21

**Authors:** Seu-Hwa Chen, June-Horng Lue, Yung-Jung Hsiao, Shu-Mei Lai, Hsin-Ying Wang, Chi-Te Lin, Ya-Chin Chen, Yi-Ju Tsai

**Affiliations:** 1 Department of Anatomy and Cell Biology, School of Medicine, College of Medicine, Taipei Medical University, Taipei, Taiwan; 2 Department of Anatomy and Cell Biology, College of Medicine, National Taiwan University, Taipei, Taiwan; 3 Department of Nursing, Central Taiwan University of Science and Technology, Taichung, Taiwan; 4 Graduate Institute of Biomedical and Pharmaceutical Science, College of Medicine, Fu Jen Catholic University, New Taipei City, Taiwan; University of Pittsburgh School of Medicine, UNITED STATES

## Abstract

In this study, we investigated temporal changes in galanin receptor type 2 (GalR2) expression in NF200-, galanin-, neuropeptide Y (NPY)-, and neuronal nitric oxide synthase (nNOS)-like immunoreactive (LI) dorsal root ganglion (DRG) neurons after median nerve chronic constriction injury (CCI), and the effects of GalR2 on c-Fos expression in the cuneate nucleus (CN). Double immunofluorescence labeling methods were used to appraise changes in GalR2 expression in NF200-LI, galanin-LI, NPY-LI, and nNOS-LI DRG neurons after CCI. The von Frey assay was used to assess the efficiency of intraplantar administration of saline, M871 (a GalR2 antagonist), or AR-M1896 (a GalR2 agonist) on neuropathic signs of rats with CCI. The effects of alterations in c-Fos expression were assessed in all treatments. The percentage of GalR2-LI neurons in lesioned DRGs increased and peaked at 1 week after CCI. We further detected that percentages of GalR2-LI neurons labeled for NF200, galanin, NPY, and nNOS significantly increased following CCI. Furthermore, M871 remarkably attenuated tactile allodynia, but the sensation was slightly aggravated by AR-M1896 after CCI. Consequentially, after electrical stimulation of the CCI-treated median nerve, the number of c-Fos-LI neurons in the cuneate nucleus (CN) was significantly reduced in the M871 group, whereas it increased in the AR-M1896 group. These results suggest that activation of GalR2, probably through NPY or nitric oxide, induces c-Fos expression in the CN and transmits mechanical allodynia sensations to the thalamus.

## Introduction

The cuneate nucleus (CN) receives innocuous tactile and proprioceptive signals from forelimb areas through primary Aβ afferent fibers and relays the information to the contralateral thalamus [1–5]. Following median nerve injury, the CN also plays a role in transmission of neuropathic pain signaling, which is partly mediated by changes in neuropeptide expression [1, 6–9]. Previous studies showed that median nerve injury leads to significant increase in neuropeptide Y (NPY) and neuronal nitric oxide synthase (nNOS) in the lesioned side of the CN and cervical dorsal root ganglion (DRG) neurons [6, 8, 10]. Results of pharmacological and morphological studies revealed that synthesis of nitric oxide potentiated NPY release from injured median nerve terminals and subsequently evoked c-Fos expression in the CN [7, 10, 11]. The expressions of the proto-oncogene *c-fos* and its protein product c-Fos have been widely used as a neuronal marker of pain following noxious stimulation [12, 13]. Furthermore, microinjection of NPY receptor antagonist ameliorated median nerve injury-induced tactile hypersensitivity and decreased c-Fos expression in the CN [7]. In addition, a previous study showed that galanin is dramatically induced in the injured side of the CN following median nerve injury [8]; however, detailed information about galanin immunolabeling in cervical DRG neurons after median nerve injury is not available.

Galanin, a 29–30 amino acid neuropeptide, is widely distributed throughout the nervous system. It affects pain threshold and has developmental and trophic effects [14, 15]. Galanin is thought to play only a minor role in nociception under normal conditions. However, it may have a critical role in modulation of nociception in neuropathic states [16–18]. In normal circumstances, galanin is expressed at low levels in rat lumbar DRG neurons, which are predominantly small-diameter C fibers [19–21]. Following sciatic nerve injury, galanin is upregulated in lumbar DRG neurons [19, 22–24]. Both pro- and anti-nociceptive effects have been attributed to galanin, probably related to the activation of different receptors [25, 26]. In fact, three galanin receptors (GalR) have been cloned so far: GalR1, GalR2, and GalR3 [23]. A pharmacological study demonstrated that GalR1 and/or GalR3 activation induced an anti-nociceptive effect, while activation of GalR2 plays a pro-nociceptive role [27]. Although it is evident that galanin and its receptors are involved in nociceptive and neuropathic pain, the impact of median nerve injury on alterations in GalR2-like immunoreactive (GalR2-LI) neurons in the DRG and their associations with NPY and nNOS expressions remain to be determined.

Therefore, in this report, we first assessed temporal changes in the amounts of galanin- and GalR2-LI neurons in the DRG following median nerve chronic constriction injury (CCI). We focused on characterizing whether GalR2-LI neurons are also associated with galanin, NPY, or nNOS following CCI. To further assess the role of GalR2 in median nerve CCI-induced neuropathic pain, we inspected neuropathic behavior signs and electrical stimulation-induced c-Fos expression in the CN following intraplantar administration of the GalR2 antagonist, M871, or the agonist, AR-M1896.

## Materials and methods

### Animal preparations

All animal experimental protocols were carried out in accordance with the UK Animals (Scientific Procedures) Act, 1986, and were scrutinized and approved by the National Science Council Committee as well as the Animal Center Committee, College of Medicine, National Taiwan University, Taiwan (IACUCA approval no. 20120407). The use of animals followed ethical guidelines from the International Association for the Study of Pain [28]. Male, Sprague-Dawley rats weighing 200–250 g were used in this study and were housed in separate cages at a temperature- (24 ± 1°C) and humidity-controlled (50%-60%) room with a 12:12-h light/dark cycle. The animals had access to food and water *ad libitum*.

### Surgery

Anesthesia was induced by an intraperitoneal (i.p.) injection of a mixture of 25 mg/kg Zoletil and 10 mg/kg xylazine. Under a dissecting microscope, the median nerve was surgically cleaned of its surrounding tissues at the level of the elbow just proximal to its entering between the two heads of the pronator teres muscle. The nerve was constricted. In the CCI group, four loose ligatures (4.0 chromic gut) were tied around the nerve, and the wound was sutured [7, 29, 30]. Animals were divided into a control group (naïve, *n* = 6) and a CCI group for 1 (*n* = 6), 2 (*n* = 4), 3 (*n* = 4), and 4 weeks (*n* = 4). In addition, in the median nerve transection (MNT) group, the nerve was ligated with silk (5.0 silk), and about a 2-mm segment of its distal end was removed [31], and survived for 1 week (*n* = 5).

### Immunocytochemistry for morphological examination

The above-described animals were re-anesthetized with a mixture of Zoletil/xylazine (25/10 mg/kg, i.p.) and perfused with 150 ml of Ringer’s solution (containing 1% sodium citrate and sodium nitrite at 4°C) via the ascending aorta, followed by Bouin fixative (acetic acid: formaldehyde: picric acid = 1:5:15 at 22°C). After that the C6 DRGs were dissected and post-fixed in the same fixatives overnight, and then embedded in paraffin mixtures. Serial sections were cut at a 7-μm thickness with a Reichert-Jung histomicrotome 820 (Buffalo, NY, USA), mounted on glass slides, and dried on a hot plate (37°C). Slides containing sections were deparaffinized in xylene, and rehydrated in decreasing ethanol concentrations to distilled water before antigen retrieval (RHS-1, Milestone, Sorisole-Bergamo, Italy, 100°C) in 0.01 M sodium citrate buffer (pH 6). The retrieved sections were treated with 0.5% H_2_O_2_ for quenching endogenous peroxidase activity, and then blocked with 5% normal horse serum (NHS, GibcoBRL, Grand Island, NY, USA) in phosphate-buffered saline (PBS) for 1 h. After rinsing with PBS, the sections were incubated in a rabbit anti-galanin antibody (1:1000, Millipore, Billerica, MA, USA) or goat anti-GalR2 antibody (1:400, Santa Cruz, Dallas, TX, USA) for 48 h at 4°C. The primary antibody was diluted in 0.1 M Tris-buffered saline (TBS, pH 7.4), containing 0.2% Tween-20 and 0.1% bovine serum albumin (BSA, Sigma-Aldrich, St. Louis, MO, USA). After sections were rinsed with PBS, they were incubated in Cy3-conjugated anti-rabbit immunoglobulin G (IgG; 1:200, Jackson ImmunoResearch, West Grove, PA, USA) or FITC-conjugated anti-goat IgG (1:200, Jackson) for 2 h at room temperature. Sections were mounted with Fluoro-Gel (EMS, Hatfield, PA, USA) and evaluated with a Leica TCS SP5-laser scanning confocal microscope (Wetzlar, Hesse, Germany).

For fluorescence double-labeling, DRG sections of control and CCI1W rats were collected and treated with 0.5% H_2_O_2_, blocked with 5% normal donkey serum (NDS, GibcoBRL) in phosphate buffer (PB) for 1 h, and incubated in goat anti-GalR2 (1:200, Santa Cruz), mouse anti-NF200 (1:1600, Sigma-Aldrich), rabbit anti-galanin (1:200, Alomone, Jerusalem, Israel), anti-NPY (1:200, Peninsula, San Carlos, CA, USA), or anti-nNOS (1:300, Sigma-Aldrich) antibodies for 48 h at 4°C. The primary antibodies were diluted in 0.1 M PB (pH 7.4) containing 0.2% Triton X-100 and 5% NDS. After rinsing in PBS, sections were incubated in FITC- or Cy3-conjugated anti-goat IgG (1:100, Jackson), FITC-conjugated anti-mouse or Cy3-conjugated anti-rabbit IgG (1:200, Jackson) for 2 h at room temperature. Sections were mounted and evaluated with a Leica TCS SP5-laser scanning confocal microscope. This evaluation included the selection of a laser gain setting used to image GalR2 + NF200, GalR2 + galanin, GalR2 + NPY, and GalR2 + nNOS double-labeling. Individual fluorescence images of Cy3 and FITC were captured and merged using Leica LAS AF software for further quantification.

### GalR2 agonist and antagonist on behavioral signs

One week before the median nerve CCI, rats were acclimatized and drilled for 3 days on all behavioral tests, followed by a baseline measurement of the tests at 1 day before CCI. Behavioral tests were repeated once every 2 days for 1 week following CCI. Five days after the CCI, rats displayed mechanical hypersensitivity, and were further given an intraplantar injection of 30 μl saline, M871 (Tocris Bioscience, Bristol, UK, 0.1 μM/paw) (1.2x10^-8^ mmol/kg), or AR-M1896 (Tocris Bioscience, 0.1 μM/paw) (1.2x10^-8^ mmol/kg) [27] (each group, *n* = 10). The behavioral tests were assessed 15 min before and at 30, 60, 90, and 120 min after drug application. All behavioral assessments were obtained by an investigator blinded to the treatment groups.

Mechanical allodynia was evaluated by means of von Frey filaments [32]. von Frey filaments (Semmes-Weinstein Monofilaments, North Coast Medical, Gilroy, CA; USA) of different bending forces, including 0.6, 1.0, 1.4, 2.0, 4.0, 6.0, 8.0, 10.0, 15.0, and 26.0 g, were used to examine the mechanical threshold of the rat forepaws [1, 7]. Briefly, tests were started with the smallest bending force and continued in increasing order. Each filament was applied five times in the medial surface of a forepaw; the first filament in the series that evoked withdrawal three times was regarded as the paw withdrawal threshold. Thresholds of individual rats in each group were averaged, and results are presented as the mean and standard error of the mean (mean ± SEM).

### Electrical stimulation

On the 7th day after CCI, a second dose of saline, M871, or AR-M1896 was administered 15 min prior to electrical stimulation. The animals were anesthetized intraperitoneally with a mixture of Zoletil/xylazine and the median nerve was carefully exposed and dissected free. Bipolar silver hook electrodes were placed beneath the isolated median nerve immediately proximal to the level of the elbow joint, at least 5 mm proximal to the constricted site. The exposed segment of the nerve was immersed in paraffin oil, and a 10-min pulse train of electrical stimulation was discharged with a 0.1-ms duration, 10-Hz frequency, and 0.1-mA intensity from a stimulator (Grass S88, Warwick, RI, USA) via a constant-current unit (Grass CCU1A) [1, 33]. For sham stimulation in half of the respective treatment groups, the median nerve was placed on stimulating electrodes, but no electrical stimulation was applied.

### Tissue preparation and immunohistochemistry

Two hours after electrical or sham stimulation, rats were re-anesthetized with a mixture of Zoletil (25 mg/kg) and xylazine (10 mg/kg) and perfused with 500 ml 4% paraformaldehyde in 0.1 M PB (pH 7.4). The brain stem containing the CN was harvested, post-fixed with the same fixative for 2 h, and stored in PB containing 30% sucrose. Tissue blocks were cut transversely into 30-μm-thick serial sections and divided in order into four sets. One of the four serial sections were treated with 1% H_2_O_2_ and blocked with 5% normal goat serum in 0.1 M PB containing 0.2% Triton X-100 for 2 h. They were incubated in rabbit anti-c-Fos (1:2000, Calbiochem, San Diego, CA, USA) antibody at 4°C for 48 h. After several washes, sections were processed with biotinylated goat anti-rabbit antiserum (Vector, Burlingame, CA, USA) for 2 h at room temperature, and processed with avidin-biotin-horseradish peroxidase (HRP) complex (ABC kit, Vector), and visualized with a Vector® SG Substrate Kit. Finally, they were mounted onto gelatinized slides and their images were captured with a digital camera (Nikon, D1X, Tokyo, Japan) through a light microscope to measure c-Fos-LI cells in the CN.

### Image analysis and statistical analysis

All assessments of galanin-LI, GalR2-LI, NPY-LI, nNOS-LI, NF200-LI and double-labeled neurons in C6 DRGs and c-Fos-LI neurons in the CN were undertaken by a researcher blinded to the treatments. To evaluate changes in their expression patterns, sections were inspected with a Zeiss light microscope (Oberkochen, Baden-Württemberg, Germany) or Leica TCS SP5-laser scanning confocal microscope. Images were captured with a Nikon digital camera or confocal microscope at 200x magnification. Numbers of neutral red-labeled, galanin-LI, GalR2-LI, and double-labeled (GalR2 + galanin, GalR2 + NPY, GalR2 + nNOS, GalR2 + NF200, galanin + NF200, NPY + NF200, and nNOS + NF200) neurons were measured from every 10th DRG section of a series. The percentage of double-labeled neurons was defined as the number of double-labeled neurons divided by the total number of neutral red-stained neurons (in an adjacent section) [6], and was then statistically compared using the Student’s *t*-test. The numbers of galanin-LI and GalR2-LI neurons divided by the total number of neutral red-stained neurons were defined as the percentages of galanin-LI and GalR2-LI neurons. Percentages of galanin-LI and GalR2-LI neurons in the MNT and CCI DRG at different time points were calculated and statistically compared using a two-way analysis of variance (ANOVA) and Bonferroni's post-hoc tests. Furthermore, galanin-, GalR2-, NPY-, and nNOS-LI neurons with a visible nucleus were classified as small (<32 μm in diameter), medium (32–50 μm in diameter), and large-sized (>50 μm in diameter) as described previously [34, 35]. For quantitative analysis, sections of the middle CN, which was defined as an area 0.3~0.7 mm caudal to the obex [1, 36], were collected from the entire rostrocaudal extent of the CN. Four sections were collected from the middle region of each animal. The mean number of c-Fos-LI cells in the CN was defined as the number of surveyed c-Fos-LI cells divided by the number of tissue sections, and was calculated and statistically compared with a one-way ANOVA and Bonferroni's post-hoc tests in the respective groups.

## Results

### Changes in galanin and GalR2 in DRGs after median nerve injury

**Fig 1:**
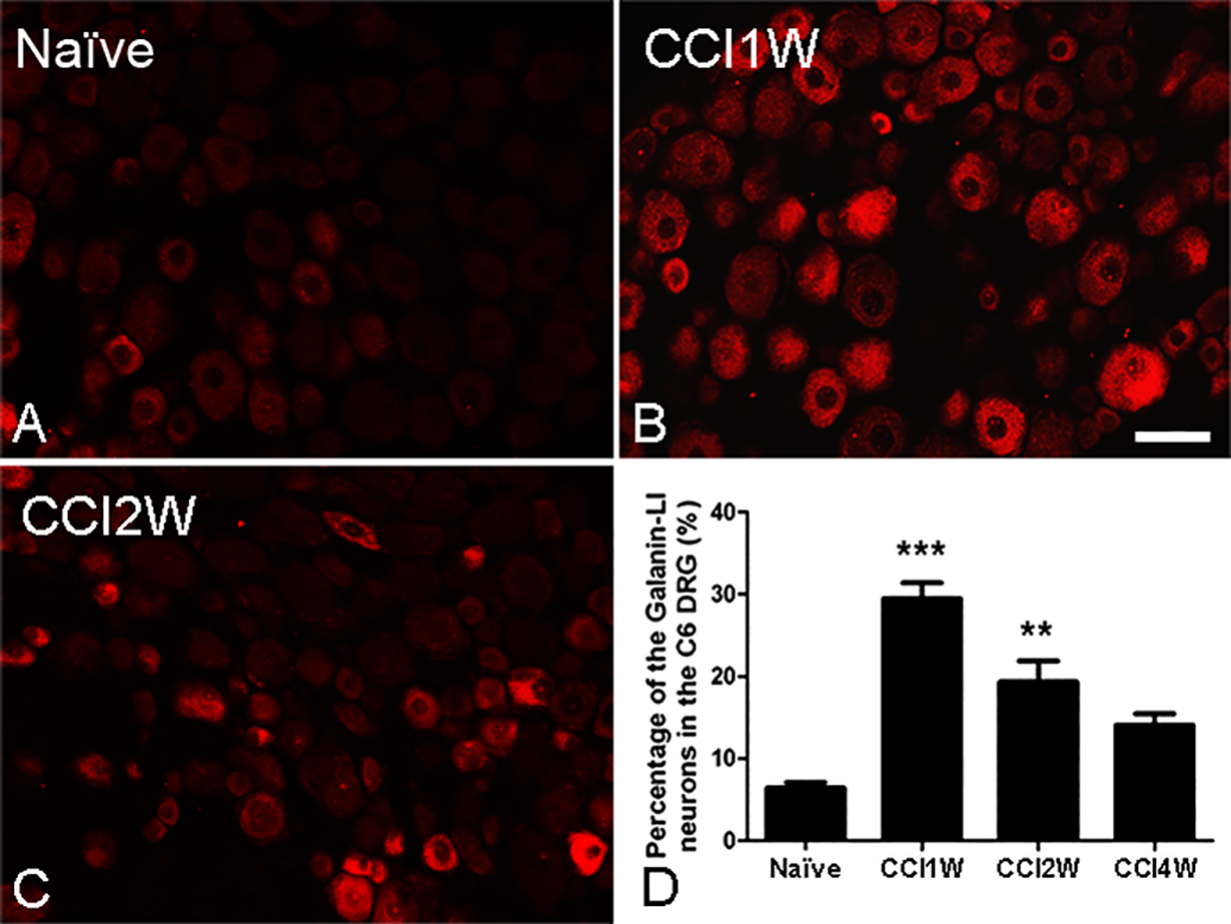
Photographs showing galanin-like immunoreactive (LI) neurons in the sixth cervical (C6) dorsal root ganglion (DRG) at various time points after chronic constriction injury (CCI). A few galanin-LI neurons were found in the C6 DRG of the naïve group (A). The number of galanin-LI neurons was detected to have increased in the DRG at 1 (B) and 2 weeks (C) following CCI. Histograms (D) display the percentage of galanin-LI neurons in the DRG at various time points following CCI. The percentage of galanin-LI neurons dramatically increased at 1 and 2 weeks and reached a peak at 1 week after CCI in the injured DRG compared to the naïve DRG (** p<0.01, *** p<0.001, compared to the naïve group. Scale bar = 50 μm).

**Fig 2:**
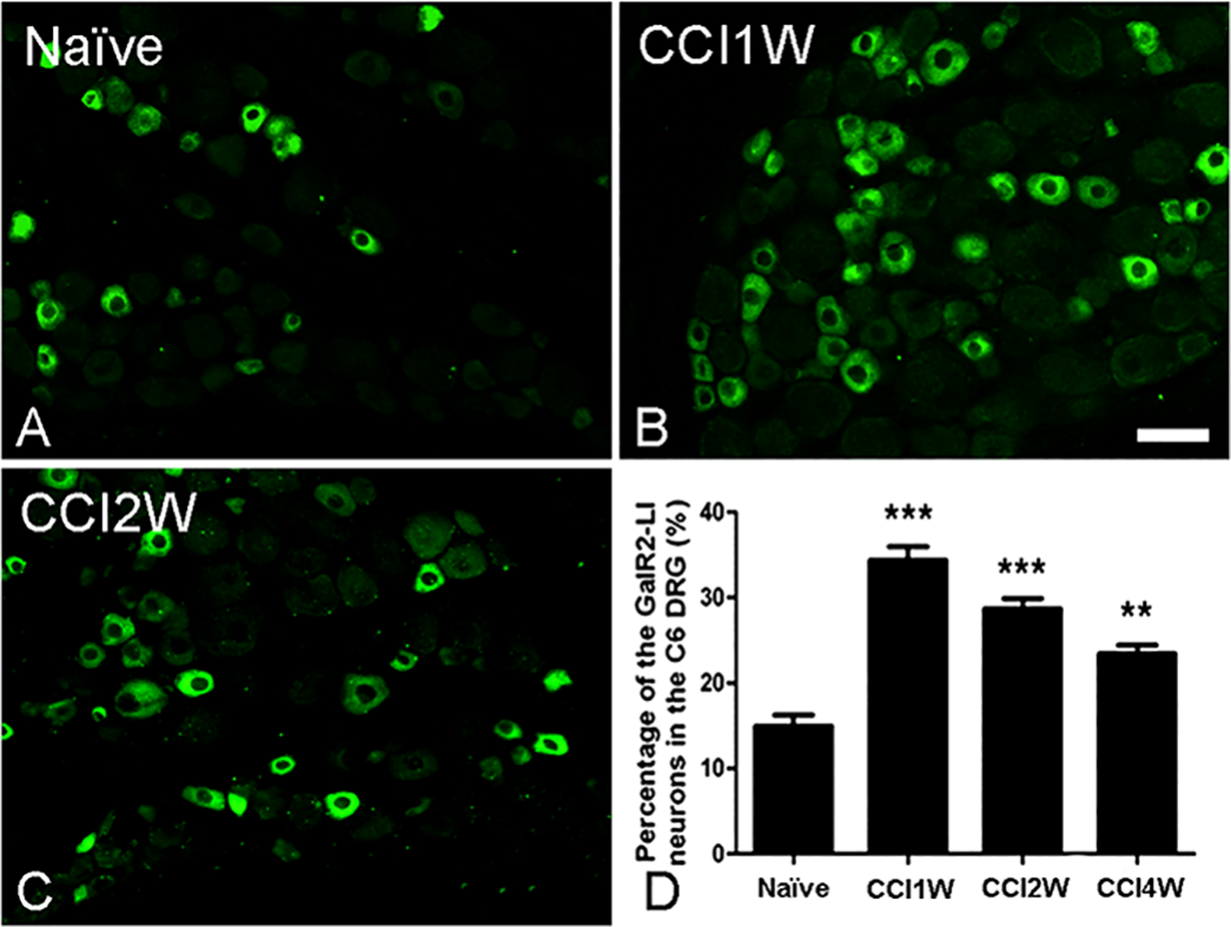
Photographs presenting galanin receptor type 2-like immunoreactive (GalR2-LI) neurons in the sixth cervical (C6) dorsal root ganglion (DRG) at various time points after median nerve chronic constriction injury (CCI). Several GalR2-LI neurons were observed in the C6 DRG of the naïve group (A). The number of GalR2-LI neurons increased in the DRG at 1 (B) and 2 weeks (C) after CCI. Histograms show the percentage of GalR2-LI neurons in the DRG at various time points following CCI (D). The percentage of GalR2-LI neurons in the DRG significantly increased after CCI and reached the highest percentage in the CCI1W group (** p<0.01, *** p<0.001, compared to the naïve group. Scale bar = 50 μm).

Using immunohistochemistry, a few galanin-LI and GalR2-LI neurons were respectively found in about 6% and 15% of surveyed neurons in control C6 DRGs, which were predominantly small-sized neurons (Figs 1A and 2A). Following median nerve CCI, amounts of galanin-LI and GalR2-LI neurons increased, peaked at 1 week after injury, and were nearly 30% of injured DRG neurons of all sizes (galanin-LI, 29.44% ± 1.96%; GalR2-LI, 34.40% ± 1.57%) (Figs 1 and 2). Additionally, in the C6 DRGs of rats 1 week after median nerve transection (MNT), we detected similar increases in both galanin-LI (29.16% ± 0.56%) and GalR2-LI neurons (31.45% ± 0.66%) (data not shown). Then, we focused on characterizing GalR2-LI neurons in C6 DRGs at 1 week after median nerve CCI.

### Characterization of GalR2-LI neurons in the DRG after median nerve CCI

**Fig 3:**
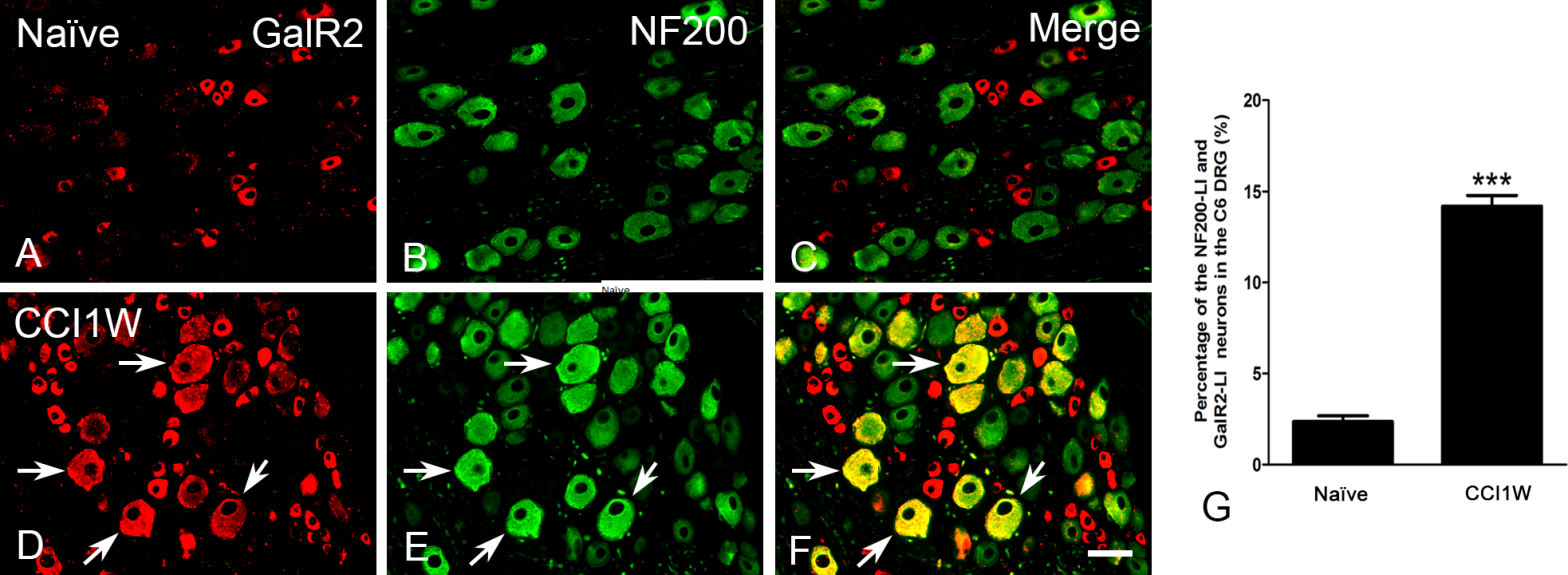
**Photographs showing double-labeling for galanin receptor type 2 (GalR2) (A, D), NF200 (B, E), and merged images (C, F) in dorsal root ganglion (DRG) neurons of the naïve (A-C) and chronic constriction injury 1 week (CCI1W) (D-F) groups.** Arrows indicate double-labeled neurons which had substantially increased in DRGs after CCI1W (F). Histograms demonstrate changes in the percentage of GalR2-like immunoreactive (LI) neurons associated with NF200 in naïve and CCI1W DRGs (G). The percentage of GalR2-LI neurons labeled for NF200 in the DRG significantly increased in the CCI1W group (*** p<0.001, Scale bar = 50 μm).

**Fig 4:**
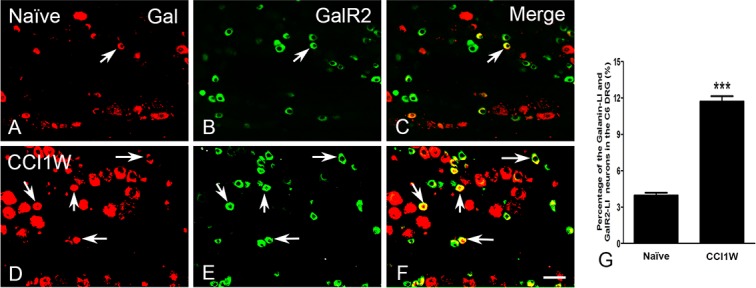
**Photographs showing double-labeling for galanin (Gal) (A, D), galanin receptor type 2 (GalR2) (B, E), and merged images (C, F) in dorsal root ganglion (DRG) neurons of the naïve (A-C) and chronic constriction injury 1 week (CCI1W) (D-F) groups.** Numbers of both Gal-like immunoreactivity (LI) (A, D) and GalR2-LI (B, E) neurons increased in the CCI1W group, and numerous GalR2-LI neurons with galanin (arrows) were found (F). Histograms show changes in the percentage of GalR2-LI neurons associated with galanin in naïve and CCI1W DRGs (G). The percentage of GalR2-LI neurons labeled for galanin in the DRG significantly increased in the CCI1W group (*** p<0.001, Scale bar = 50 μm).

**Fig 5:**
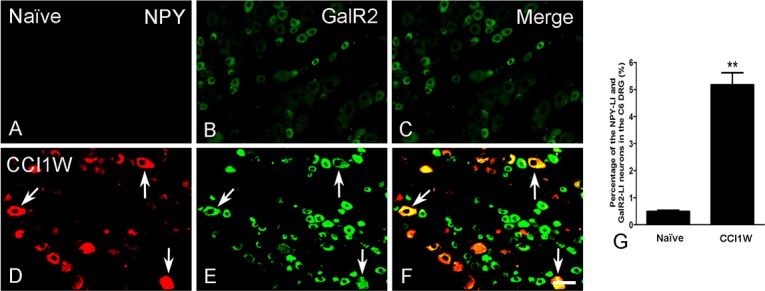
**Photographs displaying double-labeling of neuropeptide Y (NPY) (A, D) and galanin receptor type 2 (GalR2) (B, E) neurons in the dorsal root ganglion (DRG) of the naïve (A-C) and chronic constriction injury 1 week (CCI1W) (D-F) groups.** GalR2-like immunoreactive (LI) neurons were detected in both the naïve and CCI1W groups (B, E), but NPY-LI neurons were almost only found in the CCI1W group (D). The arrow indicates GalR2-LI neuron with NPY in the injured group (F). Histograms show changes in percentages of GalR2-LI neurons associated with NPY in naïve and CCI1W DRGs (G). The percentage of GalR2-LI neurons labeled for NPY in the DRG significantly increased in the CCI1W group (** p<0.01, Scale bar = 50 μm).

**Fig 6:**
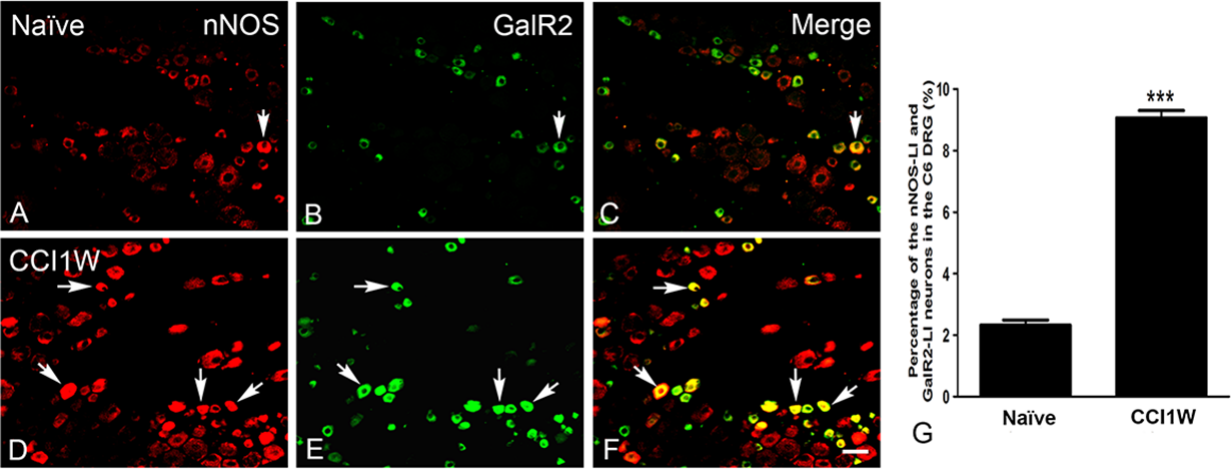
**Photographs displaying double-labeling for neuronal nitric oxide synthase (nNOS) (A, D) and galanin receptor type 2 (GalR2) (B, E), and merged images (C, F) in dorsal root ganglion (DRG) neurons of the naïve (A-C) and chronic constriction injury 1 week (CCI1W) (D-F) groups.** Arrows indicate double-labeled neurons which increased in the DRG after CCI1W (C, F). Histograms show changes in the percentage of GalR2-like immunoreactive (LI) neurons associated with nNOS in the naïve and CCI1W groups in the DRG (G). The percentage of GalR2-LI neurons coexpressed with nNOS in the DRG of the CCI1W group was significantly higher than that in the naïve group (*** p<0.001, Scale bar = 50 μm).

**Fig 7:**
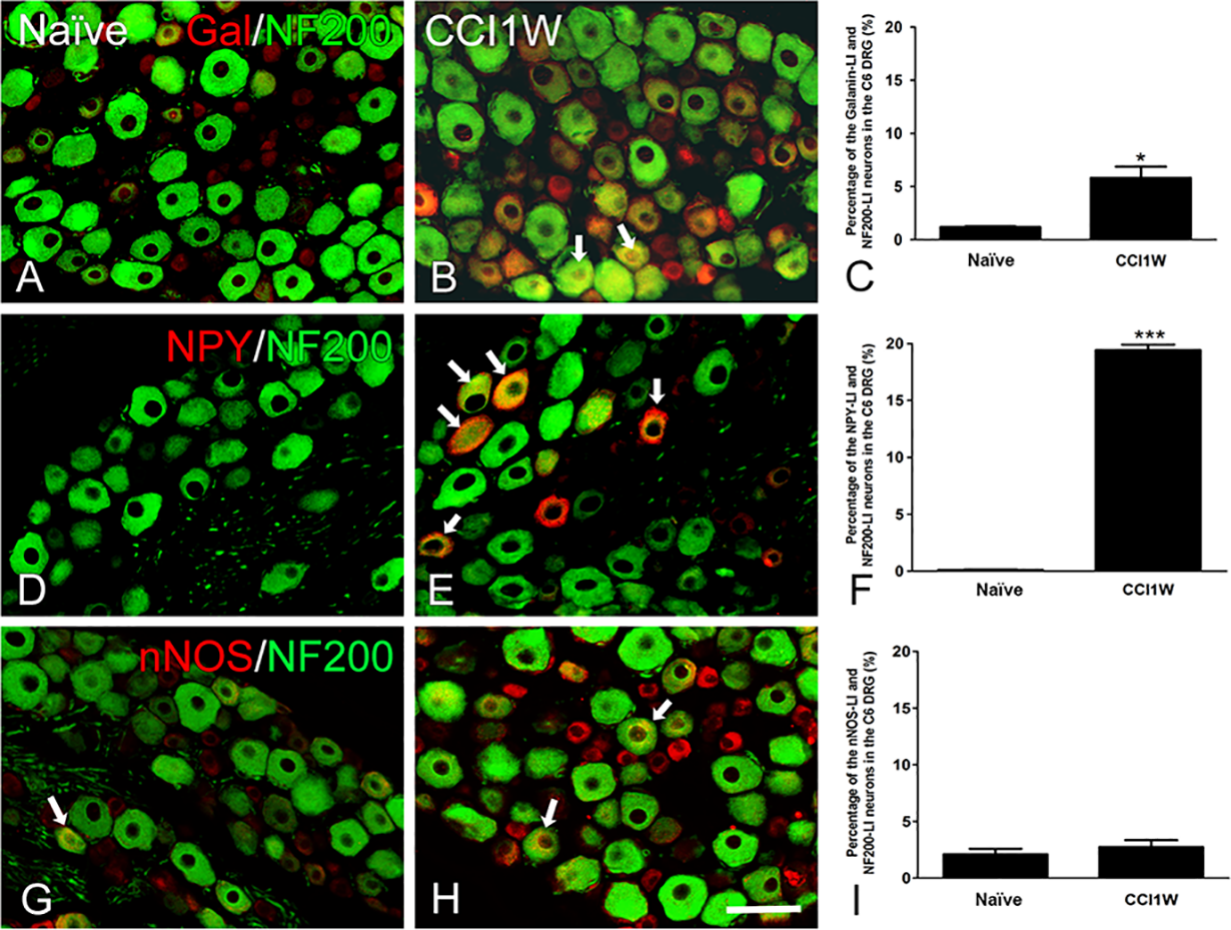
**Photographs showing galanin-like immunoreactive (Gal-LI) (A, B), neuropeptide Y (NPY)-LI (D, E), and neuronal nitric oxide synthase (nNOS)-LI (G, H) dorsal root ganglion (DRG) neurons labeled for NF200 in the naïve (A, D, G) and chronic constriction injury 1 week (CCI1W) (B, E, H) groups.** Arrows indicate double-labeled neurons. Histograms show changes in percentages of Gal-LI (C), NPY-LI (F), and nNOS-LI (I) neurons associated with NF200 in the naïve and CCI1W groups of the DRG. Percentages of Gal-LI and NPY-LI neurons labeled for NF200 in CCI DRGs were dramatically higher than those in naïve DRGs (* p<0.05, ** p<0.001), but none was detected in nNOS-LI neurons (Scale bar = 50 μm).

Under normal conditions, GalR2-LI neurons in the intact C6 DRG were almost all small-sized cells and rarely colocalized with NF200 (2.36% ± 0.32%, *n* = 3) (Fig 3), which was considered to be the A-type (large- or medium-sized) neuron marker. One week after CCI, the proportion of NF200-LI neurons in the injured DRG did not dramatically differ from that in the control group (naïve 33.91% ± 1.1% vs. CCI1W 34.95% ± 0.52%). But following CCI, increases in amounts of galanin-LI neurons (Fig 4) and proportions of NPY-LI neurons (naïve 1.20% ± 0.02% vs. CCI1W 25.82% ± 0.23%) (Fig 5) and of nNOS-LI neurons (naïve 3.57% ± 0.29% vs. CCI1W 12.00% ± 0.24%) (Fig 6) were detected in injured DRGs. Moreover, with double-labeling, percentages of GalR2-LI neurons in CCI DRGs colocalized with NF200 (naïve 2.36% ± 0.32% vs. CCI1W 14.19% ± 0.59%) (Fig 3), galanin (naïve 3.95% ± 0.23% vs. CCI1W 11.71% ± 0.44%) (Fig 4), NPY (naïve 0.49% ± 0.04% vs. CCI1W 5.17% ± 0.45%) (Fig 5), and nNOS (naïve 2.33% ± 0.16% vs. CCI1W 9.06% ± 0.24%) (Fig 6) had significantly increased compared to the control group. Percentages of galanin-LI and NPY-LI neurons labeled for NF200 in CCI DRGs were dramatically higher than those in control DRGs, but none was detected in nNOS-LI neurons (naïve vs. CCI1W, galanin: 1.20% ± 0.07% vs. 5.81% ± 1.06%; NPY: 0.11% ± 0.07% vs. 19.43% ± 0.49%; nNOS: 2.12% ± 0.48% vs. 2.75% ± 0.60%) (Fig 7).

In the naïve group, galanin- and GalR2-LI neurons in the rat DRGs were mainly small-sized (Table 1). One week after median nerve CCI, a significantly higher proportion of medium-sized galanin- and GalR2-LI neurons were observed in DRGs compared to the naïve group. There were no NPY-LI neurons detected in the naïve DRGs; by contrast, after median nerve injury, NPY-LI neurons were evidently observed, and most of them were classified as medium- and large-sized neurons. The size distribution of nNOS-LI neurons in the rat DRGs was similar between the naïve and CCI groups, and the majority of those were small-sized neurons.

**Table 1:** Number of galanin-, GalR2-, NPY- and nNOS-LI neurons in the DRG with regards to neuron sizes and experimental assignment.

Neuron size					
Total N			Small	Medium	Large
Galanin	Naïve	542	537 (99.1%)	5 (0.9%)	0 (0%)
	CCI1W*	673	543 (80.7%)	125 (18.6%)	5 (0.7%)
GalR2	Naïve	1017	1013 (99.6%)	4 (0.4%)	0 (0%)
	CCI1W*	1380	1236 (89.6%)	128 (9.3%)	16 (1.1%)
NPY	Naïve	0	0 (0%)	0 (0%)	0 (0%)
	CCI1W*	310	4 (1.3%)	235 (75.8%)	71 (22.9%)
nNOS	Naïve	312	304 (97.4%)	8 (2.6%)	0 (0%)
	CCI1W	393	373 (94.9%)	20 (5.1%)	0 (0%)

N, number of immunoreactive neurons.

Numerical data are expressed as N (%) unless otherwise indicated.

*p < 0.05 compared with the corresponding naïve group.

### Effects of the GalR2 antagonist and agonist on CCI-induced neuropathy

**Fig 8:**
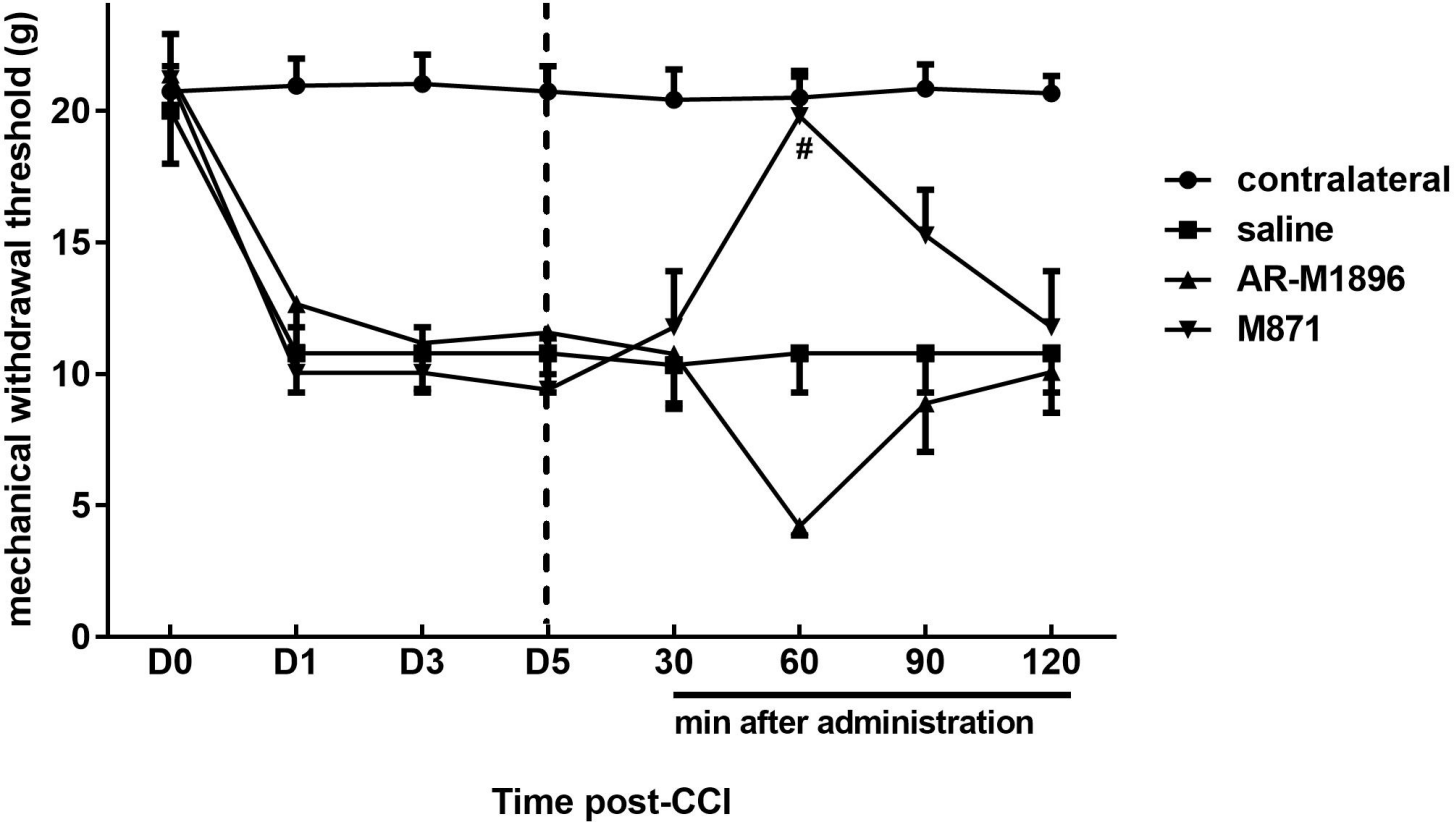
Influence of the galanin receptor type 2 (GalR2) antagonist (M871) or agonist (AR-M1896) on median nerve chronic constriction injury (CCI)-induced mechanical allodynia. The paw withdrawal threshold in response to mechanical stimuli with von Frey filaments was measured from 30 min after saline, M871, or AR-M1896 treatment in CCI rats. It is noteworthy that M871 treatment significantly suppressed mechanical allodynia at 60 min after drug administration compared to administration of saline (#P < 0.05). By contrast, CCI rats receiving AR-M1896 exhibited enhanced mechanical allodynia at 60 min compared with those receiving saline, but the difference did not reach statistical significance. The dotted line indicates the time of drug administration.

After CCI of the median nerve, rats displayed lower von Frey withdrawal threshold values on days 1~5 (Fig 8). To examine the role of GalR2 in neuropathic pain behavior, 5 days after the CCI operation, rats were given an intraplantar injection of saline, M871 (a GalR2 antagonist), or AR-M1896 (a GalR2 agonist). After M871 treatment, rats developed an increased withdrawal threshold to mechanical stimulation at 60~120 min after application. Compared to the saline group, the mechanical sensitivity was significantly relieved by M871 but slightly aggravated by AR-M1896 only at 60 min (Fig 8).

**Fig 9:**
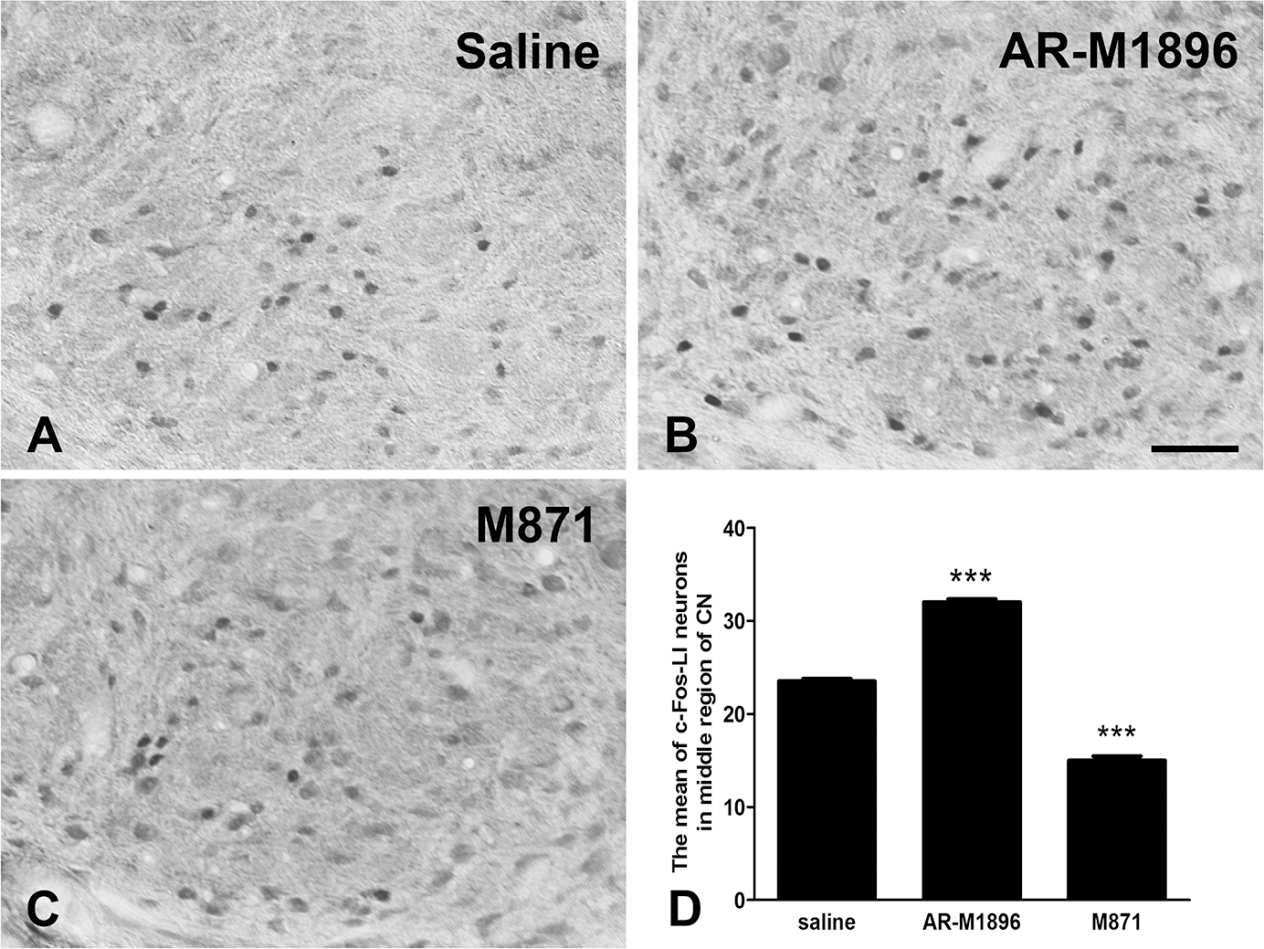
**Photographs showing c-Fos-like immunoreactive (LI) neurons in the middle region of the cuneate nucleus (CN) following unilateral electrical stimulation of the injured nerve at 1 week after median nerve chronic constriction injury (CCI) in the saline (A), AR-M1896 (B), and M871 (C) groups.** Histograms show that the mean number of c-Fos-LI neurons in the M871 treatment group was significantly less than that in the saline treatment group (*** p<0.001) (D). In contrast to the M871 group, the numerous c-Fos-LI neurons in the AR-M1896 group were significantly higher than those in the saline group (Scale bar = 50 μm).

On day 7 after CCI, rats that were treated on day 5 were given another dose of saline, AR-M1896, or M871 15 min prior to electrical stimulation to examine the effects of drug applications on c-Fos expression in the CN (Fig 9). As previously described, in all CCI rats, numerous c-Fos-LI neurons were exclusively detected in the stimulated group but not in the unstimulated group. Following electrical stimulation of the CCI-treated median nerve, many c-Fos-LI neurons in the CN were found in the saline group (Fig 9A). Interestingly, the number of c-Fos-LI neurons had increased in the AR-M1896 group (Fig 9B), but had decreased in the M871 group (Fig 9C). Compared to the saline group (23.5 ± 0.29 cells), there were a significant increase in the number of c-Fos-LI neurons in the AR-M1896 group (32 ± 0.32 cells) and a significant decrease in the M871 group (15 ± 0.45 cells) (Fig 9D).

## Discussion

The present study revealed that median nerve injury upregulated amounts of galanin-LI and GalR2-LI neurons in injured DRGs. By immunofluorescence double-labeling following median nerve CCI, percentages of GalR2-LI neurons co-expressing NF200, galanin, NPY, and nNOS immunoreactivity dramatically increased in injured DRGs. Treatment with the GalR2 antagonist, M871, ameliorated CCI-induced mechanical hypersensitivity, but it was exaggerated by the GalR2 agonist, AR-M1896. Moreover, the number of c-Fos-LI neurons in the CN was reduced by M871 treatment and was increased by AR-M1896 administration after electrical stimulation of the CCI median nerve. The functional significance of an increase in the number of GalR2-LI neurons in the DRG after CCI is discussed below.

After injury to the median nerve, the number of galanin-LI neurons in the injured DRG significantly increased and was similar to previous reports [20, 37–39]. Of note, this study is the first to demonstrate temporal changes in galanin and GalR2 expressions in the DRG after median nerve CCI, and both increased as the peak was reached at 1 week after injury. Moreover, 1 week after MNT, the number of GalR2-LI neurons had also remarkably increased in the injured DRG. This result differs from previous studies which showed downregulation of GalR2 mRNA in the lumbar DRG after sciatic nerve transection [39, 40], but was comparable to prior reports showing increases in GalR2 mRNA and immunoreactivity in the ipsilateral DRG after peripheral tissue inflammation [40, 41]. The reason for this variance is not clear but is likely due to different methods employed; i.e. in situ hybridization [39, 40] versus immunohistochemistry, or different segmental DRGs investigated could possibly have resulted in this difference. Taken together, both CCI and transection of the median nerve may incur increased percentages of GalR2-LI neurons in C6 DRGs.

The present study showed that the percentages of GalR2-, galanin-, and NPY-LI neurons labeled for NF200 in the injured DRG were significantly increased after median nerve CCI, suggesting that some of the above-mentioned injury-induced elements were expressed by medium- or large-sized DRG neurons and may subsequently give rise to Aα/β primary afferent fibers to the CN [1]. It has been shown that profound changes in galanin [8, 42] and NPY [7, 8] expressions in the CN following nerve injury are involved in the neuropathic pain mechanism. Characterization of upregulated GalR2-LI neurons in the injured DRG at 1 week after CCI was used with immunofluorescence double-labeling for galanin, NPY, and nNOS. Of note, this study is the first to show that amounts of GalR2-LI neurons containing galanin, NPY, and nNOS in CCI DRGs were significantly higher than those in control DRGs. Burazin and Gundlach reported that galanin and GalR2 mRNA levels increased in the ipsilateral facial motor nucleus after facial nerve crush, and both displayed a similar temporal expression pattern; therefore, the study suggests that the GalR2 may act as the active galanin “autoreceptor” during nerve injury [43]. Then the functional role of injury-increased galanin activation of GalR2 was thought to process pro-nociception [27, 44]. Moreover, the present study also provides novel evidence that many GalR2-LI neurons were labeled for NPY in the median nerve CCI DRG, but not in the control group. Recent studies elucidated that median nerve injury-induced NPY participates in development of tactile, but not thermal, hypersensitivity [7, 30]. This study further provides for the first time that the percentage of GalR2-LI neurons colocalized with nNOS in CCI DRGs was significant higher than that in the control DRG. Our previous results showed that median nerve injury caused upregulation of the number of nNOS-LI neurons in the cervical DRG and CN [10, 11]. The nNOS-LI neurons in the DRG were predominantly found to be small-sized and were previously regarded to function not only in mechanical allodynia but also in thermal hyperalgesia after nerve injury via its synthesizing the product NO [11, 45, 46]. Taken together, we speculate that increased proportions of galanin-, NPY- and nNOS-containing GalR2-LI neurons in the DRG may make neuropathic pain processing much more complicated; however, the exact functional role of each type of neurons needs to be clarified in the coming studies.

The present intraplantar application of the GalR2 antagonist, M871, and agonist, AR-M1896, significantly attenuated and aggravated mechanical allodynia, respectively, in CCI rats. These behavioral findings are consistent with previous studies which demonstrated that activating GalR2 augmented nerve injury-induced nociceptive behaviors [41, 47]. These data indicate that GalR2 is a major candidate for mediating the peripheral excitatory effect of galanin, which is strongly supported by the fact that high percentages of peripheral digital nerves were labeled for galanin and GalR2 [41]. Comparing the present double-labeling results, the percentage of GalR2-LI neurons labeled for NPY or nNOS after CCI was upregulated. Thus, it is reasonable to assume that the increased GalR2-LI neurons play an important role in mechanical allodynia development after CCI.

This study is also the first to demonstrate that the number of c-Fos-LI cells in the CN after electrical stimulation of the injured nerve was reduced by M871 treatment and increased by AR-M1896 application. The present results differ from a previous study which found that AR-M1896 reduced the glutamate-induced c-Fos protein in primary neural hippocampal cells [48]. Although the reason for this discrepancy remains uncertain, it might have been due to differences in the dose applied or nuclei examined. Moreover, c-Fos-LI cells were only detected in the CN with electrical stimulation of the CCI median nerve, but not in the CN without stimulation. Based on our observation that GalR2-LI neurons contained NPY and/or galanin, one possible explanation is that activation of GalR2 modulated injury-induced NPY or galanin release from primary afferent terminals to induce c-Fos expression in the CN by electrical stimulation. This speculation is supported by an earlier study that reduced NPY expression and c-Fos induction are observed in the stimulated side of the CN following electrical stimulation of the transected median nerve [7]. Furthermore, as to the functional significance of c-Fos induction in the CN, it was previously reported that the density of c-Fos-LI cells is positively correlated with the magnitude of mechanical allodynia [7, 30]. Molander *et al*. reported that all c-Fos-LI cells in the spinal cord dorsal horn are neurons but not astroglia or microglia after electrical stimulation of the transected sciatic nerve [49]. In previous studies, we found that pronounced c-Fos expression is induced in the thalamic projection neurons of the CN by electrical stimulation of injured median nerve in neuropathic rats [1, 7, 31]. It is therefore proposed that after electrical stimulation of the injured median nerve, the induced expression of c-Fos in the CN thalamic projection neurons may exert an ascending influence on the thalamus for propagating the neuropathic pain signals. Taken together, injection of AR-M1896, via binding to GalR2, activates the G_q/11_ and subsequent Ca^2+^-phospholipase C-dependent protein kinase C pathway [48, 50], which may lead to NPY and/or galanin release from primary afferent terminals and c-Fos expression in the thalamic projection neurons of the CN following electrical stimulation of the injured median nerve, and then provide the ascending thalamic transmission of neuropathic pain signals. On the contrary, application of M871, which competes with galanin for binding to GalR2 [27, 51], intercepts the above signal transduction cascade.

Although many nerve injury studies have been carried out in hindlimb nerve models, in particular the sciatic nerve, differences in nociceptive circuitry between forelimbs and hindlimbs of rats were evident [52, 53]. Therefore, experimental results obtained from studies in hindlimbs of rats may not be generalized and applicable to forelimbs. In addition, the majority of human peripheral nerve injuries involve the upper limbs, and median nerve injury is commonly encountered as a result of laceration, fracture-associated stretch and contusion, compression, and injection injuries [54, 55]. Patients with median nerve injury may experience exquisite pain to tough over the affected sites, with even a light touch triggering a disproportionate pain reaction [54, 56]. In this study, we observed prominent changes in the mechanical withdrawal threshold to light touch by von Frey filaments in a rat model of median nerve CCI. Thus, the animal model applied herein seems to simulate human upper limb neuropathy and will be useful in studying for clinically relevant disorders.

## Supporting information

S1 File Spreadsheet containing the dataset used in this study. (XLSX)

## Abbreviations

C6: sixth cervical
CCI: chronic constriction injury
CN: cuneate nucleus
DRG: dorsal root ganglion
GalR: galanin receptor
GalR2: galanin receptor type 2
GalR2-LI: GalR2-like immunoreactive
MNT: median nerve transection
nNOS: neuronal nitric oxide synthase
NPY: neuropeptide Y
PB: phosphate buffer
PBS: phosphate-buffered saline
